# Non-pharmaceutical therapy for post-stroke shoulder-hand syndrome: Protocol for a systematic and network meta-analysis

**DOI:** 10.1097/MD.0000000000020527

**Published:** 2020-06-05

**Authors:** Qiang Gao, Huaili Nie, Chunyan Zhu, Naifeng Kuang, Xiaoyu Wang, Yiqian Chen, Xiao Zhang, Dali Zheng, Qing Xia, Tao Yin, Limin Pan, Liangzhen Xie

**Affiliations:** a Taian City Central Hospital; b Taian Sanatorium of Shandong Province, Taian; c Nongken Jiansanjiang People Hospital of Heilongjiang Province, Jiansanjiang; d Harbin Medical University; e Heilongjiang University of Chinese Medicine, Second Affiliated Hospital; f Heilongjiang University of Chinese Medicine, First Affiliated Hospital, Harbin, China.

**Keywords:** network meta-analysis, non-pharmaceutical therapeutic strategy, post-stroke shoulder hand syndrome, protocol

## Abstract

**Background:**

Shoulder-hand syndrome (SHS) is a common complication in post-stroke patients. SHS has a large impact on patients and their families, communities, healthcare systems and businesses throughout the world. Non-pharmaceutical therapy for post-stroke SHS is the most common treatment in clinical practice, but their effectiveness is still unclear. The aim of this study is to assess the effect and safety of non-pharmaceutical therapeutic strategies for post-stroke SHS.

**Method:**

We will search 3 in English and 4 in Chinese languages electronic databases regardless of publication date or language. We will include randomized controlled trials (RCTs) assessing the effect of any non-pharmaceutical therapy for post-stroke SHS. Primary outcomes will be any effective instrument for post-stroke SHS. Two authors will independently assess the risk of bias by using Cochrane tool of risk of bias. We will perform network meta-analysis in random effects model to estimate the indirect and mixed effects of different therapeutic strategies by R-3.5.1 software. We will assess the confidence in cumulative evidence by Grading of Recommendations Assessment, Development and Evaluation.

**Results:**

This study will be to assess the effect and safety of non-pharmaceutical therapy for post-stroke SHS.

**Conclusions:**

This study will assess the effect of different non-pharmaceutical therapeutic strategies for post-stroke SHS and provide reliable evidence for the choice of treatments.

**Systematic review registration:** PROSPERO (CRD42019139993).

## 1 Introduction

Shoulder-hand syndrome (SHS) defined as clinical syndromes of pain, hyperalgesia, joint swelling, and limitations in the range of motion, is a common complication in post-stroke patients.^[1–2]^ SHS was also called reflex sympathetic dystrophy (RSD) or complex regional pain syndrome (CRPS) type I and usually appeared in 2 to 36 weeks after stroke, with its reported prevalence ranging from 1.5% to 70%.^[3–5]^ SHS occurs commonly in over 60 years old patients between 2 weeks to 3 months after stroke, and more commonly in men than women.^[6]^ On account of insidious and slow clinical symptoms, SHS usually results in patients’ neglect and delays in optimal treatment so that it not only aggravates the patients stroke, but also is difficult to be cured in clinical practice. Meanwhile, post-stroke SHS brings the huge physical and mental shock for the patients and burdens for medical staff and patients’ families.^[7–8]^ Thus, post-stroke SHS is a common and serious public health problem, and effective therapeutic strategies are required for the prevention.

In clinical practice, the therapeutic strategies for post-stroke SHS should be focused on multidisciplinary comprehensive treatment.^[9]^ Standardized and systematic treatment for post-stroke SHS improved limb function and clinical effects. Various kinds of treatments are used to alleviate post-stroke SHS, including physical therapy, drug therapy, and traditional therapy. At present non-pharmaceutical therapy for post-stroke SHS is the most common treatment. Several studies showed that physical therapy and acupuncture produced positive changes in affective aspects of pain in patients with post-stroke SHS.^[10–12]^

To our knowledge, non-pharmaceutical therapy for post-stroke SHS is the most common treatment in clinical practice, but the effectiveness of non-pharmaceutical therapeutic strategy was still unclear. Although some meta-analyses had been published,^[13–16]^ there was lack of multiple comparisons in clinical studies so that it was more difficult to make the ideal choice. Network meta-analyses was a way to assess the effects of more than 2 treatments for the same condition by direct and indirect comparisons.^[17]^ Therefore, we conducted the first network meta-analyses that comprehensively integrated the eligible RCT. Meanwhile, we intended to assess the effect of different therapeutic strategies for post-stroke SHS and provide reliable evidence for the choice of treatments.

## 2 Methods

### 2.1 Objectives and registration

This review will be to assess the effect and safety of non-pharmaceutical therapy for post-stroke SHS. This review protocol has been registered in the PROSPERO that is the International Prospective Register of Systematic Reviews (registered number: CRD42019139993).

### 2.2 Inclusion criteria

#### 2.2.1 Types of studies

The inclusion criteria of studies will be RCTs in this systematic review regardless of publication status and language.

#### 2.2.2 Types of participants

In our study, participants diagnosed with post-stroke shoulder-hand syndrome will be included regardless of their age, sex, or race.

#### 2.2.3 Types of interventions

In our study, we will evaluate the efficacy of non-pharmaceutical therapy for patients with post-stroke shoulder-hand syndrome in clinical practice, including acupuncture, manual therapy, massage, traction, yoga and exercise, mobilization, training, surgery etc.

#### 2.2.4 Types of outcome measures

In our study, primary outcomes will be any effective instrument for SHS, for example, visual analogue scale, Fugl–Meyer Assessment scale etc. Secondary outcomes will include functional comprehensive assessment, quality of life, and adverse events.

### 2.3 Search methods for the identification of studies

We will search 3 English and 4 Chinese-language electronic databases regardless of publication date or language, including Cochrane Library, Medline, EMBASE, Chinese Biomedical Database, China National Knowledge Infrastructure, Chinese VIP Information, and Wangfang Database. We will conduct different strategies for 7 electronic databases based on trial terms (random, trial, group), symptom terms (shoulder-hand syndrome, stroke complication, complex regional pain syndrome), treatment terms (acupuncture, manual therapy, massage, traction, Yoga and exercise, mobilization, training, surgery).

### 2.4 Data collection

**Figure 1:**
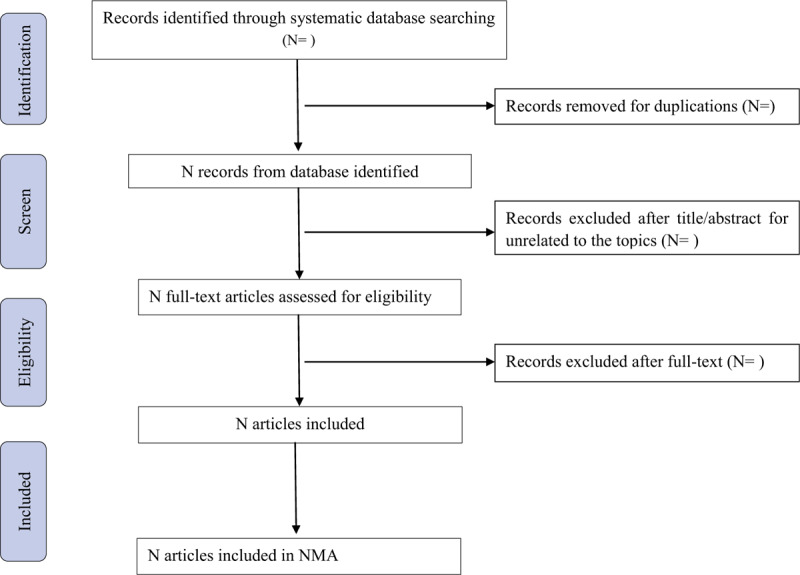
Flow chart of study selection.

The titles and abstracts will be selected and identified by 2 independent reviewers (GQ and NHL). The full text will be assessed. If there are any disagreements, they will be discussed and resolved by an experienced reviewer (PLM). All articles will be stored in a separate database. The 2 reviewers (GQ and NHL) will independently assess all eligible studies according to the inclusion criteria. The Preferred Reporting Items for Systematic Reviews and Meta-Analyses flow chart will be used to report the reasons for excluding and including eligible studies. The study selection procedure will be shown in the Preferred Reporting Items for Systematic Reviews and Meta-Analyses flow chart (Fig. 1).

### 2.5 Assessment of the risk of bias

The risk of bias for all included studies will be assessed by 2 independent authors (GQ and NHL) by the Cochrane tool of risk of bias (V.5.1.0). Any disagreements will be settled by a third experienced reviewer (YT). The following aspects will be assessed for risk of bias: selection, performance, detection, attrition, reporting, and other factors. All included studies will be classified into 3 categories: high risk, low risk, and unclear level of risk.

### 2.6 Data synthesis and statistical analysis

#### 2.6.1 Intervention comparisons: direct

We will calculate mean difference or standardized mean difference with their 95% confidence intervals for continuous variables. We will calculate risk ratios or odds ratio with their 95% confidence intervals for categorical variables. We will perform standard pairwise meta-analysis in random effects model by R-3.5.1 software where heterogeneity of interventions permits. For insufficient or missing data, we will contact the authors by e-mail or phone as much as possible.

#### 2.6.2 Intervention comparisons: indirect and mixed

We will conduct network meta-analysis in random effects model to estimate the indirect and mixed effects of therapeutic strategies for SHS by package netmeta version 1.13 of R-3.5.1 software. We will assess the inconsistency between direct and indirect comparisons by node-splitting method. We will summarize the rank of each treatment for different outcomes and evaluate by surface under cumulative ranking curve.

#### 2.6.3 Subgroup and sensitivity analysis

We will test heterogeneity by standard Chi-squared statistic and Higgins I^2^ statistic. We will perform subgroup analyses in order to explore the differences in the age, sex, methodological quality, subtypes, and race/ethnicity if there is high heterogeneity in the included studies. To evaluate the robustness of the pooled results, we will perform a sensitivity analysis to assess the impact of the trials with a high risk of bias. We will compare the findings to determine whether lower quality studies should be excluded based on the size of the sample, the strength of the evidence and its impact on the overall scale of effectiveness.

#### 2.6.4 Reporting biases

We will use funnel plots to identify whether there will be the potential for small study bias if there are sufficient studies. If there are asymmetry of funnel plots that suggest possible small study effects, the results of analysis will be explained cautiously.

#### 2.6.5 Confidence in cumulative evidence

The Grading of Recommendations Assessment, Development and Evaluation will be used to evaluate the quality of evidence on outcomes. The quality of the body of evidence will be assessed based on 5 factors, including study limitations, effect consistency, imprecision, indirectness, and publication bias. The studies will be assigned 1 of 4 possible ratings: very low, low, moderate, or high.

## 3 Ethics and dissemination

Ethical approval is not appropriate, on account of this protocol for network meta-analysis. In our study there will be no patients recruited, and no data gathered from patients. This review will be disseminated by the approach of peer-reviewed publications.

## Author contributions

PLM and YT developed the study protocol. GQ and NHL developed the search strategy with supervision of XLZ. GQ and NHL will scan the included studies, extract the data and assess the risk of bias with supervision of PLM and YT. QG and XQ will perform data analysis with supervision of PLM and YT. All authors (GQ, NHL, ZCY, KNF, WXY, CYQ, ZX, ZDL, XQ, YT, PLM and XLZ) will contribute to data interpretation. GQ, NHL, ZCY, KNF, WXY, CYQ, ZX, ZDL, XQ, YT, PLM and XLZ drafted and revised the manuscript. All authors have read and approved the final version of the manuscript.

**Conceptualization:** Limin Pan, Tao Yin.

**Data curation:** Qiang Gao, Huaili Nei, Qing Xia.

**Formal analysis:** Qing Xia.

**Funding acquisition:** Qiang Gao, Liangzhen Xie.

**Methodology:** Huaili Nei, Limin Pan, Tao Yin, Liangzhen Xie.

**Software:** Qiang Gao, Qing Xia.

**Supervision:** Limin Pan, Tao Yin, Liangzhen Xie.

**Visualization:** Qiang Gao, Huaili Nei, Chunyan Zhu, Naifeng Kuang, Xiaoyu Wang, Yiqian Chen, Xiao Zhang, Dali Zheng, Qing Xia, Limin Pan, Tao Yin, Liangzhen Xie.

**Writing – original draft:** Qiang Gao, Huaili Nei, Chunyan Zhu, Naifeng Kuang, Xiaoyu Wang, Yiqian Chen, Xiao Zhang, Dali Zheng, Qing Xia, Limin Pan, Tao Yin, Liangzhen Xie.

**Writing – review & editing:** Qiang Gao, Huaili Nei, Chunyan Zhu, Naifeng Kuang, Xiaoyu Wang, Yiqian Chen, Xiao Zhang, Dali Zheng, Qing Xia, Limin Pan, Tao Yin, Liangzhen Xie.
